# Inhibitory Activity of Pyrroloisoxazolidine Derivatives against *Chlamydia trachomatis*

**DOI:** 10.1155/2021/8889247

**Published:** 2021-03-13

**Authors:** Min Ni, Shunxin Xu, Ziyi Liu, Yin Xue, Wenxia Xie, Shengju Yang, Lingyan Liu, Xiaofeng Bao

**Affiliations:** ^1^School of Pharmacy, Nantong University, Nantong 226001, China; ^2^Department of Dermatology and Venereology, Affiliated Hospital of Nantong University, Nantong 226001, China; ^3^State Key Laboratory of Elemento-Organic Chemsitry, Nankai University, Tianjin 300071, China; ^4^Key Laboratory of Inflammation and Molecular Drug Target of Jiangsu Province, Nantong University, Nantong 226001, China

## Abstract

The obligate intracellular bacterium *Chlamydia trachomatis* is a group of worldwide human pathogens that can lead to serious reproductive problems. The frequent clinical treatment failure promoted the development of novel antichlamydial agents. Here, we firstly reported a group of pyrroloisoxazolidine-inhibited *C. trachomatis* in a dose-dependent manner *in vitro*. Among them, compounds 1 and 2 exhibited the strongest inhibitory activity with IC_50_ values from 7.25 to 9.73 *μ*M. The compounds disturbed the whole intracellular life cycle of *C. trachomatis*, mainly targeting the middle reticulate body proliferation stages. Besides, the compounds partially inhibited the chlamydial infection by reducing elementary body infectivity at high concentration. Our findings suggest the potential of pyrroloisoxazolidine derivatives as promising lead molecules for the development of antichlamydial agents.

## 1. Introduction

*Chlamydia trachomatis* is the most prevalent bacterial pathogen that leads to sexually transmitted diseases (STD) in the world [1, 2]. The serotypes D-K are associated with urogenital tract infection, resulting in serious complications including ectopic pregnancy, abortion, infertility, and pelvic inflammatory disease in women [2, 3]. The invasive serovars L1-L3 can cause lymphogranuloma venereum (LGV) that can lead to serious disseminated infections of the lymphatic system [2, 3]. In the United States, among the 2.3 million STD cases reported to the Centers for Disease Control and Prevention (CDC) in 2017, *C. trachomatis* infection remained the most common condition reported. More than 1.7 million cases were diagnosed as *C. trachomatis* infection, with 45 percent among 15- to 24-year-old females. In addition, the serovars A-C infect the ocular mucosa causing trachoma, the leading cause of preventable infectious blindness in many of the undeveloped countries [4, 5].

*Chlamydia trachomatis* is an obligate intracellular bacterium characterized by a highly specialized biphasic developmental cycle, morphologically switch between the extracellular infectious but nonproliferative elementary body (EB) and the intracellular replicative but noninfectious reticulate body (RB). The infection is initiated by EB binding to and invading the host cell. Within the following 6-8 h, internalized EB starts to transcript early genes and differentiates into RB inside the specialized vacuole inclusion. Subsequently, replicative RB divides by binary fission before redifferentiating back to infectious EB. Most chlamydial developmental cycles are complete in 40-72 hours when EBs are released from the host cell by either inclusion extrusion or cell lysis [1, 6, 7].

Currently, uncomplicated *C. trachomatis* infections are commonly treated with either a single dose of azithromycin or doxycycline twice a day for at least 7 days [8, 9]. Unfortunately, as the development of antibiotic-resistant bacteria, relapsing symptoms and treatment failures are frequently found after treatment with these first-choice antibiotics, resulting in *Chlamydia* persistence or long-term infection [10–12]. During the past few decades, plenty of efforts have been focused on vaccine development. However, no effective vaccine is available to prevent *C. trachomatis* infections up to date [13–15]. All these negative situations raise the pressing need to search for novel antichlamydial agents from natural materials or synthesized chemicals [16–25]. Isoxazolidine derivatives are a group of chemicals that possess a variety of biological and pharmaceutical activities including neuroactive, antioxidant, antibacterial, antifungal, and antiretroviral properties [26–30]. In this study, we firstly reported the antichlamydial activity of our previously synthesized eighteen pyrroloisoxazolidines via the hydroamination cyclization-[2+3]-cycloaddition with homopropargylic amines and nitrones using metal catalysts AgOAc [31]. We revealed the fact that these compounds affected the intracellular proliferation period of *C. trachomatis*, mainly on the middle RB proliferation stages.

## 2. Materials and Methods

### 2.1. Pyrroloisoxazolidine Derivatives

The pyrroloisoxazolidine derivatives 1-18 were synthesized and isolated as previous [31]. Compounds were dissolved in DMSO and were diluted to the appropriate concentrations using culture medium. To eliminate the interference of possible aggregates in diluted samples that might cause nonspecific inhibition against *Chlamydia* [32, 33], culture medium containing individual compound was centrifuged for 30 min at 12700 rpm (18213 g) at room temperature. The supernatant was then used to assess the antichlamydial activity as described in the following.

### 2.2. Chlamydia Strains and Culture Condition

*Chlamydia trachomatis* serovars D (strain UW-3/CX) and L2 (strain 434/Bu) were purchased from ATCC. HeLa cells purchased from ATCC were routinely cultured in DMEM supplemented with 10% heat-inactivated FBS (Sigma-Aldrich) and 10 *μ*g/mL gentamicin at 37°C in a humidified atmosphere of 5% and were used for chlamydial propagation and cell culture experiments. EBs were purified by Renografin density gradient and stored in sucrose-phosphate-glutamate (SPG) buffer at -80°C [20]. A mouse polyclonal anti-MoPn (*C. muridarum* strain Nigg II) antibody, which was produced in our laboratory and cross-reacting with *C. trachomatis* D and L2, was used as a primary antibody for both stains detection [20].

### 2.3. Inclusion Body Immunofluorescence Staining

Cell monolayers seeded onto coverslips in a 24-well plate were infected with *C. trachomatis* at a multiplicity of infection (MOI) of 0.2 inclusion-forming units (IFU) per cell. Unless noted, individual compound was added into culture medium at indicated concentrations simultaneously at the time of inoculation. 0.5% DMSO was used as negative control and 11.25 *μ*M tetracycline (Sigma-Aldrich) as positive control [20]. For *C. trachomatis* D, an additional centrifugation (900 × g at room temperature for 1 h) was applied to facilitate the infection. At 36 hours postinfection (hpi), infected cells were fixed by precooled methanol and then subjected to sequential staining processed with a primary antibody and a FITC-conjugated secondary antibody. Evans blue was used as cytoplasm counterstain. Images were shot by an Olympus IX51 fluorescence microscope.

### 2.4. Infectious Progeny Inhibition Assay

Cells monolayers seeded in 48-well plate were infected and treated with chemicals as above. After incubation for 36 hours, infected cells were washed twice, scraped off, and lysed by sonication to release the infectious progeny EBs. Then the lysates were used to reinfect fresh cell monolayers grown in 96-well plate following 1 : 10 serial dilution. The inclusion counts were obtained by immunofluorescence staining as above to calculate the number of infectious progeny EBs in each sample. Percent inhibition was calculated based on EB counts in compound-treated samples relative to DMSO-treated negative control.

### 2.5. WST-1 Cell Viability

Uninfected HeLa cells were cultured with 32 *μ*M (the highest concentration used in antichlamydial analysis) of individual compound or 0.5% DMSO for 48 h. After 10 *μ*L of WST-1 reagent was added, cells were incubated for an additional 1 h, and absorbance at 450 nm was recorded. The viability of compound-treated cells was calculated by the absorbance relative to that of DMSO control setting as 100% [22].

### 2.6. Host Cell Cytotoxicity

Uninfected HeLa cells were cultured with indicated concentration of individual compound or 0.5% DMSO. Forty-eight hours later, cells were fixed with methanol and stained with Evans blue for morphology or DAPI for nuclear integrity. Otherwise, at 48 h later, cells were detached and enumerated by a hemacytometer [20].

### 2.7. Host Cell Pretreatment Assay

Cells seeded in 48-well plate were incubated with 2, 8, or 32 *μ*M individual compound at 37°C for indicated times. After twice washing with SPG to remove residual compound, cells were infected with EBs at an MOI of 0.2. At 36 hpi, the inclusion count in each well was determined by immunofluorescence staining as above.

### 2.8. Elementary Body Pretreatment Assay

*Chlamydia trachomatis* L2 EBs suspended in SPG at 4 x 10^5^ IFU/mL were exposed to 2, 8, or 32 *μ*M individual compound at 37°C or 4°C for indicated times. EBs then were washed twice to eliminate the residual chemical and were immediately loaded onto HeLa cell monolayers at an MOI of 0.2. The infective EB count was measured by quantifying inclusion numbers at 36 hpi using immunofluorescence staining as above.

### 2.9. Later Treatment Assay

HeLa cells seeded in 48-well plate were infected with C. *trachomatis* L2 at an MOI of 0.2 and were treated with 2, 8, or 32 *μ*M of compound at 0, 2, 12, and 24 hpi. At 36 hpi, cells were collected, and the production of infectious progeny EBs was determined as described above. Percent inhibition was calculated based on progeny EB count in compound-treated samples relative to DMSO-treated negative control.

### 2.10. Withdrawal Assay

HeLa cells seeded in 48-well plate were infected with C. *trachomatis* L2 at an MOI of 0.2 and simultaneously treated with 2, 8, or 32 *μ*M of compound. At 2, 12, 24, and 36 hpi, infected cells were washed twice to remove residual inhibitor, and fresh medium was added. At 36 hpi, the production of infectious progeny EBs and percent inhibition data were determined as above.

### 2.11. Period Incubation Assay

HeLa cells seeded in 48-well plate were infected with *C. trachomatis* L2 at an MOI of 0.2 and treated with 2, 8, or 32 *μ*M of compound during 0-2, 2-12, 12-24, and 24-36 h period. At 36 hpi, the production of infectious progeny EBs and percent inhibition data were determined as above.

### 2.12. Statistical Analysis

Data from the three independent experiments were presented as mean ± standard deviations (SD) and were subjected to pairwise Student's *t*-tests. *p* < 0.05 and *p* < 0.01 are considered statistically significant and statistically highly significant, respectively. The IC_50_ values, defined as the concentration of compound at which the generation of infectious progeny EBs was reduced by 50% relative to negative control, were calculated by nonlinear regression from percent inhibition data using the GraphPad Prism 5 software, and presented as mean (95% confidence interval).

## 3. Results

### 3.1. Pyrroloisoxazolidines Inhibited *C. trachomatis* in a Dose-Dependent Manner

The antichlamydial activity of eighteen synthetic pyrroloisoxazolidines was screened by determining their inhibitory effect on the generation of infectious progeny EBs at concentration doubly increased from 1 to 32 *μ*M against *C. trachomatis* D and L2 [22, 34]. The IC_50_ values were calculated from percent inhibition data and presented in Table 1. All the compounds inhibited both *C. trachomatis* with IC_50_ values lower than 32 *μ*M for at least one tested strain. Among them, compounds 1 and 2 showed the best inhibitory activity with similar IC_50_ values slightly lower than 10 *μ*M for both strains. For *C. trachomatis* D, compounds 4, 5, and 7 exhibited IC_50_ values higher than 32 *μ*M, and those were compounds 9, 11, 13, 17, and 18 for *C. trachomatis* L2. All other compounds possessed IC_50_ values between 10 and 32 *μ*M.

As obligate intracellular pathogens, *C. trachomatis* relied on the host cells for survival and replication. To exclude the possibility of cytotoxicity that disturbing chlamydial growth and displayed as compounds' antichlamydial activity, the cytotoxic effect of compounds on host cells was assessed using the WST-1 method by treatment of uninfected HeLa cells with 32 *μ*M of individual compound, the highest concentration used in our antichlamydial analysis. As presented in Table 1, most compounds did not affect the proliferation activity of HeLa cells, except three chemicals (6, 7, and 10) that weakly decreased the cell viability to about 90% of DMSO control. Because compounds 1 and 2 have the highest inhibitory activity but not cytotoxicity, they were chosen for further antichlamydial mechanism analysis.

**Figure 1:**
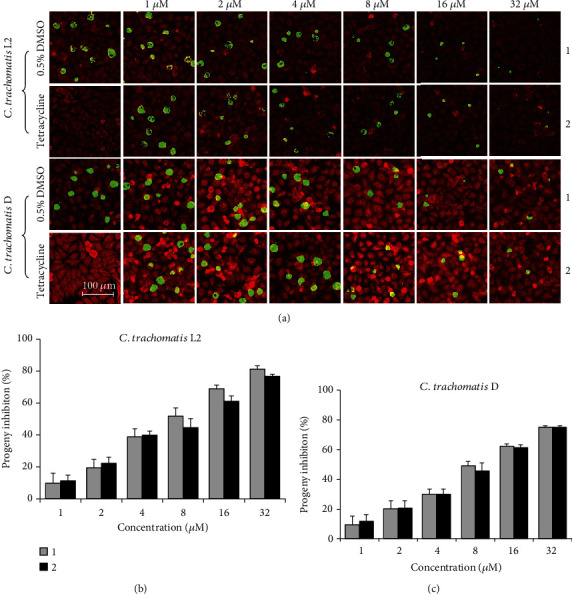
Inhibition effect of compounds 1 and 2 on *C. trachomatis*. HeLa cells were infected with *C. trachomatis* L2 or D at an MOI of 0.2 in the presence of 0.5% DMSO, 11.25 *μ*M tetracycline, or various concentrations of individual compound. Cells were either fixed or lysed for determination of infectious progeny EBs at 36 hpi. (a) Representative immunofluorescence microscopy images (scale bar = 100 *μ*m). Inclusions were stained with a polyclonal anti-MoPn antibody that cross-reacts with *C. trachomatis* L2 and D (green), and cells were counterstained with Evans blue (red). (b, c) The percent inhibition of production of infectious progeny EBs was calculated based on data in compound-treated samples relative to DMSO-treated control. Data are presented as mean ± SD of three independent assays.

To understand how the compounds inhibited *C. trachomatis*, the direct inhibitory effect of compounds 1 and 2 was evaluate using immunofluorescence staining by culturing *C. trachomatis* L2-infected cells containing individual compound. Compared with the negative control (0.5% DMSO), both compounds inhibited the formation (number) and growth (size) of *C. trachomatis* L2 inclusions in a dose-dependent manner (Figure 1(a)). At the highest concentration of 32 *μ*M, inclusions were few and tiny, analogous to the positive control tetracycline (final concentration, 11.25 *μ*M) [19]. In addition, the reduction on the production of progeny EBs was consistent with that of the inclusion number and size (Figure 1(b)). The direct inhibitory effect was also analyzed for *C. trachomatis* D. Happened as expected, changes in inclusion size and number and the progeny production that response to both chemicals are highly similar to those observed for *C. trachomatis* L2, with slightly weaker effect (Figures 1(a) and 1(c)).

**Figure 2:**
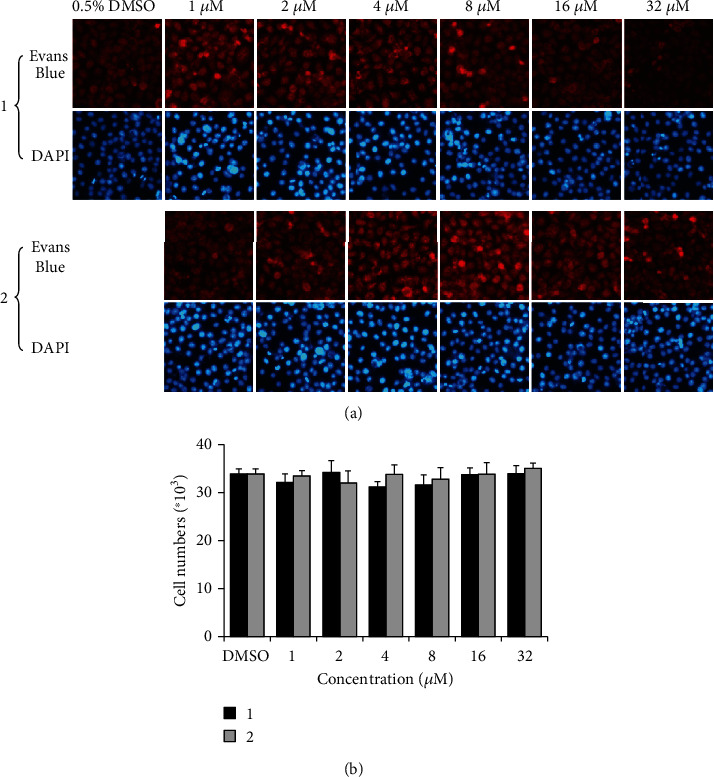
Lack of cytotoxicity of compounds 1 and 2 on HeLa cells. Uninfected HeLa cells were cultured with medium containing various concentrations of individual compound or 0.5% DMSO for 48 h. (a) The cellular morphology was stained with Evans blue, and cell nucleus was stained with DAPI. (b) The cells were enumerated by a hemacytometer. Data are presented as mean ± SD of three independent assays.

As shown in Figure 1(a), the morphology of infected HeLa cells was well preserved when treated with compound. To further verify that compounds 1 and 2 are not toxic to host cells, the uninfected HeLa cells were treated with individual compound for 48 h, and then their morphology, nuclear integrity, and cell proliferation were measured [20]. No substantial cytotoxic activity on host cells was observed from morphological features by Evans blue staining, nuclear integrity by DAPI staining, and cell proliferation by counting cell numbers (Figure 2). Coupled with the WST-1 data (Table 1), these findings verified that compounds 1 and 2 do not affect host cells, boosting the conclusion that the antichlamydial activity of compounds is not through host cell toxicity.

### 3.2. Pyrroloisoxazolidines Weakly Attenuated the EB Infectivity

*Chlamydia trachomatis* has a unique EB/RB biphasic life cycle, which offers the infection steps and the intracellular proliferation period for therapeutic intervention. To explore whether the compounds affected the chlamydial infection steps, the HeLa cells or EBs were pretreated with compound prior to infection, and then the influence on EB entry efficiency was analyzed by counting inclusion numbers. Due to the relative easier for culture and shorter developmental cycle, *C. trachomatis* L2 is commonly represented as a laboratory model for *C. trachomatis* in cell culture research and is picked for our subsequent mechanism study.

**Figure 3:**
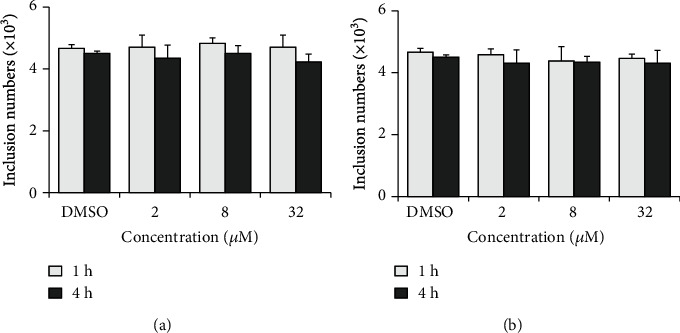
Host cells were not involved in the compounds' antichlamydial activity. HeLa cells were incubated with 2, 8, or 32 *μ*M compound 1 (a) or 2 (b) at 37°C for 1 or 4 h. After washes, cells were infected with EBs. At 36 hpi, the inclusion was counted as described in “Materials and Methods.” Data are presented as mean ± SD of three independent assays.

**Figure 4:**
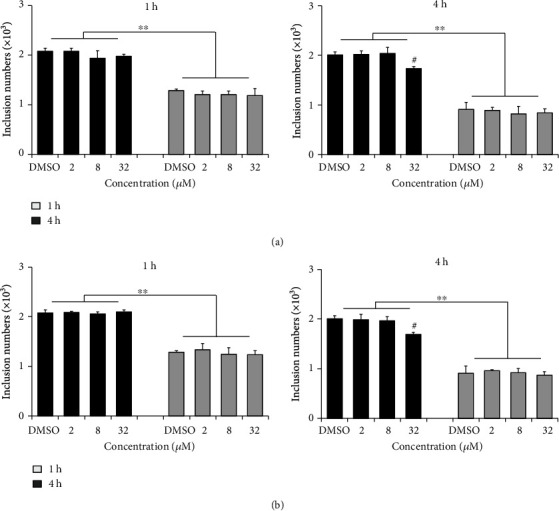
Compounds 1 and 2 weakly attenuated the EB infectivity. *Chlamydia trachomatis* L2 EBs were incubated with 2, 8, or 32 *μ*M compound 1 (a) or 2 (b) at 37°C or 4°C for 1 or 4 h. After washes, EBs were used to infect HeLa cell. At 36 hpi, the inclusion was counted as described in “Materials and Methods.” Data are presented as mean ± SD of three independent assays. ∗∗*p* < 0.01, compared 4°C with 37°C by Student's *t*-tests. ^#^*p* < 0.05, compared with each DMSO control sample by Student's *t*-tests.

As shown in Figure 3, pretreatment of HeLa cells with 2, 8, and 32 *μ*M of compound 1 or 2 prior to infection for 1 h did not reduce the formation of chlamydial inclusions. The inclusion number did not change even extending the incubation time to 4 hours, indicating that both compounds did not affect the host cell during infection process. Because EBs suffered a loss of infectivity at 37°C in SPG buffer containing 0.5% DMSO (Figure 4), 4°C EB pretreatment was supplemented to the physiological temperature. When EBs were pretreated with 2, 8, and 32 *μ*M of compound for 1 h, the EB counts that enter into host cells did not change at both temperatures (Figure 4). However, if the EBs were pretreated with chemical for 4 h, the EB entry efficiency slightly dropped at the highest 32 *μ*M for both compounds at 4°C, revealing a mild interaction between the compound and EB that weakly attenuated the EB infectivity.

### 3.3. Pyrroloisoxazolidines Disturbed the Chlamydial Intracellular Life Cycle

**Figure 5:**
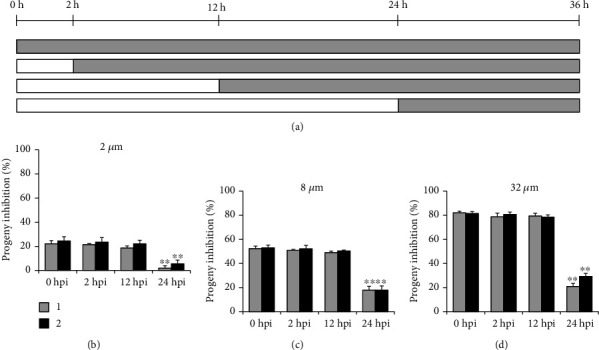
Later treatment assay. (a) HeLa cells infected with *C. trachomatis* L2 were treated with 2, 8, or 32 *μ*M individual compound at 0, 2, 12, or 24 hpi. The production of progeny EBs was determined at 36 hpi. (b–d) Percent inhibition of progeny EBs was calculated based on data in compound-treated samples relative to DMSO-treated control. Data are presented as mean ± SD of three independent assays. ∗∗*p* < 0.01, compared with each 0 hpi positive control sample by Student's *t*-tests.

Since the chlamydial infection process was not the primary target of the compounds, the intracellular proliferation cycle was analyzed. Firstly, the later treatment assay was used that the compound was added to infected cells at different times postinoculation, and then the production of infectious progenies was quantified at 36 hpi (Figure 5(a)). As shown in Figures 5(b)–5(d), compared with 0 hpi sample (positive control, the cells were treated with compound for whole incubation period), the inhibitory activity for both compounds was maintained when chemical was added before 12 hpi. However, the inhibitory effect was dramatically dropped if the compound was added at 24 hpi, when the suppression activity was weak but still maintaining a substantial effect.

**Figure 6:**
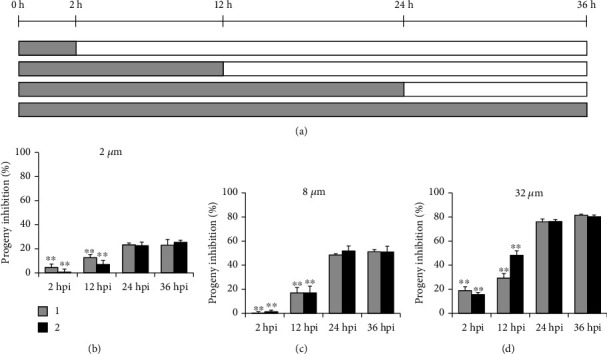
Withdrawal assay. (a) HeLa cells infected with *C. trachomatis* L2 were simultaneously treated with 2, 8, or 32 *μ*M individual compound. The compound was removed at 2, 12, 24, or 36 hpi, and the production of progeny EBs was determined at 36 hpi. (b–d) Percent inhibition of progeny EBs was calculated based on data in compound-treated samples relative to DMSO-treated control. Data are presented as mean ± SD of three independent assays. ∗*p* < 0.05, ∗∗*p* < 0.01, compared with each 36 hpi positive control sample by Student's *t*-tests.

Secondly, the withdrawal assay was performed that the compound was added simultaneously with inoculation and then was removed at different times postinfection. The production of infectious progenies was quantified at 36 hpi to evaluate the effect of different treatment scheme (Figure 6(a)). As lower concentrations of 2 and 8 *μ*M (Figures 6(b) and 6(c)), compared with 36 hpi sample (positive control, the cells were treated with compound for whole infection period), the inhibitory capacity for both compounds was maintained when chemical was removed after 24 hpi. At highest 32 *μ*M (Figure 6(d)), the inhibition effect slightly dropped from approximately 80% to 75% at 24 hpi. For all concentrations tested, the inhibitory efficiency for both compounds was significantly reduced if the chemical was removed before 12 hpi. The earlier the compound was removed, the weaker inhibitory effect was obtained.

**Figure 7:**
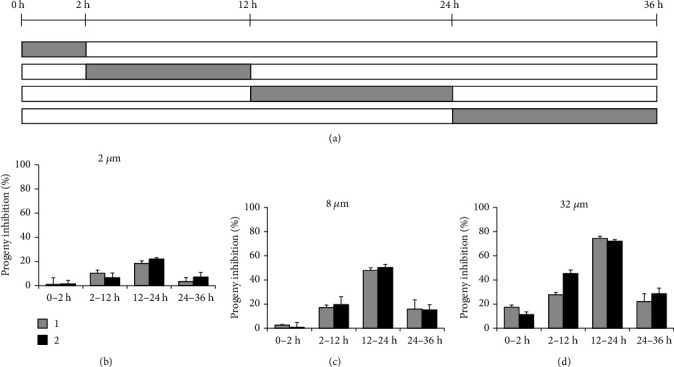
Period incubation assay. (a) HeLa cells infected with *C. trachomatis* L2 were treated with 2, 8, or 32 *μ*M individual compound for indicated times. The production of progeny EBs was determined at 36 hpi. (b–d) Percent inhibition of progeny EBs was calculated based on data in compound-treated samples relative to DMSO-treated control. Data are presented as mean ± SD of three independent assays.

Finally, the period incubation assay was applied that the infected cells were treated with compound for indicated time periods, and then the production of infectious progenies was quantified at 36 hpi (Figure 7(a)). For all concentrations (Figures 7(b)–7(d)), both compounds almost lost their antichlamydial activity with 0-2 h treatment except the highest 32 *μ*M. They possessed the strongest inhibitory effect at 12-24 h period close to the full-time positive control. Besides, both 2-12 h and 24-36 h treatment revealed similarly partial inhibition, slightly higher for 2-12 h period.

## 4. Discussion

*Chlamydia trachomatis* is a worldwide pathogen responsible for a variety of human diseases. Although it is susceptible to several broad-spectrum antibiotics, clinical treatment failure has happened frequently. Until now, there is no effective vaccine for human use. These facts necessitate the continuous efforts to discover and develop novel nonantibiotic antichlamydial agents [16–25].

In search for antichlamydial agents from the synthesized compounds, we firstly reported that a group of pyrroloisoxazolidines inhibited *C. trachomatis* propagation. The IC_50_ values from the yield of infectious progeny EBs against *C. trachomatis* L2 and D exhibited that most of the derivatives inhibited chlamydial growth in micromolar range (Table 1). Compounds 1 and 2 showed the best inhibitory activity with IC_50_ values in the range of 7.25 to 9.73 *μ*M. The C-4 methoxyl group of phenyl on R2 position of compounds 4 and 5 was contributed to the weak antichlamydial activity on *C. trachomatis* D. Compound 16 displayed the best inhibitory activity on *C. trachomatis* D with IC_50_ value of 5.93 *μ*M, which indicated the C-4 methyl group of phenyl on both R1 and R2 favored its inhibitory activity. Compound 1 displayed the best inhibitory activity on *C. trachomatis* L2 with IC_50_ value of 7.25 *μ*M, which indicated the substitute groups on phenyl was harmful to their inhibitory activity. These derivatives also showed no selectivity in their inhibitory activity against *C. trachomatis* L2 and D, which suggested that they might inhibit both *Chlamydia* strains through the same mechanism.

WST-1 data showed that at 32 *μ*M, the highest concentration used in our antichlamydial assays, most compounds had no apparent effects on the host cell viability except compounds 6, 7, and 10 which weakly decreased the cell viability to about 90% of DMSO control (Table 1). The compounds 1 and 2, the derivatives with the best antichlamydial activity, did not show cytotoxicity using the WST-1 method. In addition, the cellular morphology, nuclear integrity, and cell proliferation data also eliminated the potential toxicity of compounds 1 and 2 that may obstruct chlamydial growth (Figure 2), easily coming to a conclusion that the antichlamydial activity of the compounds is not related to their cytotoxicity.

The developmental cycle of *C. trachomatis* begins with infection process as adhesion and entry of the EB into a host cell, relying on multiple redundant factors both on the host cell and bacteria surface [35]. Pretreatment of HeLa cells with compound 1 or 2 could not impair the EB counts that enter into cells (Figure 3), excluding the roles of host cell factors that involved in the compounds' antichlamydial activity. Lower concentration and shorter time incubation of EBs with compound 1 or 2 also did not affect the entered EB counts (Figure 4). However, when the chemical concentration was raised to 32 *μ*M and the incubation time was extended to 4 hours, the EB infectivity was weakly but statistically decreased (Figure 4). This result was verified by the period incubation assay that 32 *μ*M compound 1 or 2 possessed a weak inhibitory effect if treated at 0-2 h (Figure 7(d)), the EB binding and invading stages. These combined data suggested the potential effect of compounds on bacteria surface factors that are related to the attachment/entry of *C. trachomatis* into host cells to reduce EB infectivity, which might be partially concerned in the inhibitory effect of the compounds.

The compound pretreatment only weakly impaired EBs infectivity, while the cotreatment condition almost fully blocked chlamydial growth (Figure 1), pointing out the speculation that the compounds affected the intracellular stages of the chlamydial life cycle. According to the later treatment assay, the antichlamydial activity of compounds 1 and 2 was maintained when chemical was added before 12 hpi while it was dramatically dropped if added at 24 hpi (Figure 5), indicating the strong inhibition on the intracellular period of *C. trachomatis* after 12 hpi. Meanwhile, the withdrawal assay data revealed that the inhibitory effect of compounds 1 and 2 was relatively well preserved when chemical was removed after 24 hpi while it was significantly reduced if removed before 12 hpi (Figure 6), suggesting the strong intracellular proliferation inhibition before 24 hpi. Based on these two results, a conclusion was easily deduced that the compounds primarily affected the middle 12-24 h period of *C. trachomatis* life cycle, when the RB was replicated during this phase. This conclusion was well documented by the observation that the production of infectious progenies was significantly inhibited when the compound 1 or 2 was added during the 12-24 h period (Figure 7), which was close to the 0-36 h full-time positive control (Figures 5 and 6).

Compounds 1 and 2 kept a weak but substantial inhibitory effect when added at 24 hpi (Figure 5). These results suggested that the intracellular life cycle of *C. trachomatis* after 24 hours could be disturbed by the compounds although it was not that powerful, confirmed by the 24-36 h period incubation when the infectious progeny EBs were partially inhibited (Figure 7). Furthermore, the 2-12 h period of intracellular chlamydial growth was also partially prevented by compounds 1 and 2 (Figure 7), verified by the 12 hpi withdrawal data (Figure 6).

## 5. Conclusions

In summary, we firstly identified the antichlamydial activity of a group of pyrroloisoxazolidines in vitro. Among them, compounds 1 and 2 exhibit the greatest inhibitory activity with IC_50_ values in the range of 7.25 to 9.73 *μ*M. All combined results came to a clear conclusion that the compounds could interfere with the whole intracellular cycle of *C. trachomatis*, mainly targeting the middle RB proliferation periods. Our findings suggest the potential of pyrroloisoxazolidine derivatives as lead molecules for the development of antichlamydial agents. Additional animal studies were required to verify the compounds' antichlamydial activity in vivo.

## Figures and Tables

**Table 1:** The IC_50_ values (*μ*M) and cell viability of pyrroloisoxazolidine-treated uninfected cells. IC_50_ values are presented as the mean (95% confidence interval). ∗ indicates statistically significant (*p* < 0.05) difference of cell proliferation activity between compound-treated and DMSO-treated cells.

Entry	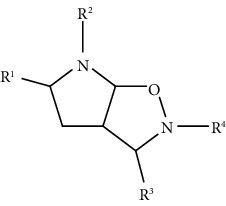				IC_50_ (*μ*M) Mean (95% confidence interval)		Cell viability (%)
	R^1^	R^2^	R^3^	R^4^	*Ct* L2	*Ct* D	32 *μ*M
1	Ph	Ph	Ph	Ph	7.25 (6.48 ~ 8.11)	9.31 (8.42 ~ 10.28)	100.92
2	Ph	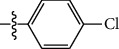	Ph	Ph	8.69 (7.59 ~ 9.93)	9.73 (8.83 ~ 10.72)	98.32
3	Ph	Ph	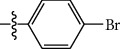	Ph	8.67 (6.40 ~ 11.73)	20.91 (17.80 ~ 24.58)	99.89
4	Ph	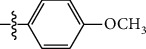	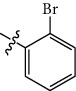	Ph	11.17 (7.49 ~ 16.64)	>32	97.49
5	Ph	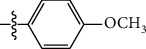	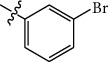	Ph	15.49 (8.04 ~ 29.87)	>32	95.17
6	Ph	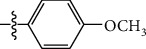	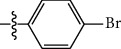	Ph	9.08 (4.66 ~ 17.68)	16.85 (12.61 ~ 22.52)	90.12∗
7	Ph	Ph	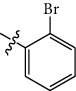	Ph	19.06 (12.84 ~ 28.30)	>32	88.26∗
8	Ph	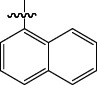	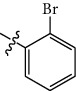	Ph	25.42 (12.62 ~ 51.22)	28.60 (18.77 ~ 43.58)	101.81
9	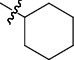	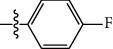	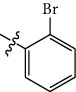	Ph	>32	19.59 (13.18 ~ 29.11)	96.51
10	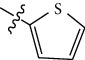	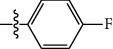	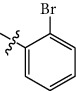	Ph	17.35 (13.02 ~ 23.10)	15.43 (12.94 ~ 18.40)	90.71∗
11	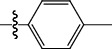	Ph	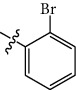	Ph	>32	23.54 (16.13 ~ 34.35)	99.70
12	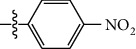	Ph	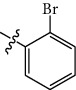	Ph	17.52 (11.07 ~ 27.73)	12.51 (9.86 ~ 15.87)	103.85
13	Ph	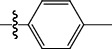	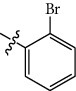	Ph	>32	16.29 (12.03 ~ 22.06)	95.73
14	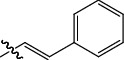	Ph	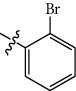	Ph	16.78 (13.61 ~ 20.70)	16.31 (12.81 ~ 20.77)	97.95
15	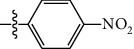	Ph	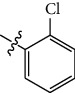	Ph	9.77 (8.45 ~ 11.30)	12.22 (7.94 ~ 18.80)	103.64
16	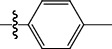	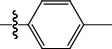	Ph	Ph	13.52 (12.86 ~ 14.21)	5.93 (4.51 ~ 7.79)	96.53
17	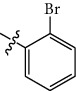	Ph	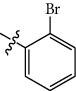	Ph	>32	27.57 (20.56 ~ 36.98)	113.63
18	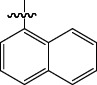	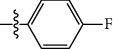	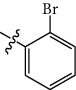	Ph	>32	20.97 (16.41 ~ 26.79)	105.86

## Data Availability

The data used to support the findings of this study are included within the article.
